# Hydrodynamics of an Electrochemical Membrane Bioreactor

**DOI:** 10.1038/srep10387

**Published:** 2015-05-22

**Authors:** Ya-Zhou Wang, Yun-Kun Wang, Chuan-Shu He, Hou-Yun Yang, Guo-Ping Sheng, Jin-You Shen, Yang Mu, Han-Qing Yu

**Affiliations:** 1 CAS Key Laboratory of Urban Pollutant Conversion, Department of Chemistry, University of Science and Technology of China, Hefei, China; 2 Jiangsu Key Laboratory of Chemical Pollution Control and Resources Reuse, Nanjing University of Science and Technology.

## Abstract

An electrochemical membrane bioreactor (EMBR) has recently been developed for energy recovery and wastewater treatment. The hydrodynamics of the EMBR would significantly affect the mass transfers and reaction kinetics, exerting a pronounced effect on reactor performance. However, only scarce information is available to date. In this study, the hydrodynamic characteristics of the EMBR were investigated through various approaches. Tracer tests were adopted to generate residence time distribution curves at various hydraulic residence times, and three hydraulic models were developed to simulate the results of tracer studies. In addition, the detailed flow patterns of the EMBR were acquired from a computational fluid dynamics (CFD) simulation. Compared to the tank-in-series and axial dispersion ones, the Martin model could describe hydraulic performance of the EBMR better. CFD simulation results clearly indicated the existence of a preferential or circuitous flow in the EMBR. Moreover, the possible locations of dead zones in the EMBR were visualized through the CFD simulation. Based on these results, the relationship between the reactor performance and the hydrodynamics of EMBR was further elucidated relative to the current generation. The results of this study would benefit the design, operation and optimization of the EMBR for simultaneous energy recovery and wastewater treatment.

Microbial fuel cells (MFCs) are devices that convert the chemical energy stored in organic/inorganic matter to electricity through bioelectrochemical reactions while using bacteria as catalysts^1^,^2^,^3^,^4^. A membrane bioreactor (MBR) consists of a filtration membrane for separating the suspended solids and a bioreactor for biodegrading the dissolved organic and inorganic constituents^5^,^6^. By coupling these two systems, novel electrochemical membrane bioreactors (EMBRs) have been developed for recovering energy from wastewater while harvesting clean water for reuse^7^,^8^,^9^,^10^,^11^. In these reactors, membrane module was served as a separator or filter between anode and cathode, sometimes could even be the cathode. The substrates were consumed by the bacteria so that electrons and protons were produced at the anode, and the treated water would be filtered by the separator and cathode, electrons reached the cathode through the electrodes and external circuit then combined with oxygen from the air or aeration and protons that diffused from anode. Compared with individual MFC or MBR, This combination would decrease the spending of reactors and brought high-quality effluent, high nutrient (especially nitrogen) removal efficiency and a certain electrical energy.

Hydrodynamic characteristics are highly significant for bioreactors because they can determine the phase distribution and residence time in the reactor^12^. Good mixing in the reactor has many benefits, such as stopping sludge or biofilm growth, removing organic compounds, and granting complete use of the reactor volume^13^,^14^. The hydrodynamics of both MFCs and MBRs have been investigated through various approaches, such as residence time distribution (RTD) analyses and/or computational fluid dynamics (CFD) simulations. Moon *et al* reported that non-ideal flows, such as tailing and channeling, would increase the power generation in a normal graphite felt disk MFC relative to a perforated graphite felt disk MFC^15^. Dekker *et al.* found that shorter HRTs brought improved the cathode performance in scaled-up and stacked MFC because the flexible membrane generated unequal effective volumes^16^. A CFD model has been developed to account for the aeration, sludge rheology and geometry of two full-scale MBRs^17^, while Sanaeepur *et al.* have adopted the CFD simulation to explore nitrate mass transfer while investigating the denitrification efficiency in an extractive MBR^18^. Because the EMBR was developed by coupling these two systems, the hydrodynamic characteristics of the EMBR should also significantly affect the mass transfers and reaction kinetics in the reactor, strongly affecting the reactor performance. To the best of our knowledge, the hydrodynamic characteristics of the EMBR remain largely unknown.

The present study aims to investigate the hydrodynamic characteristics of the EMBR. Three different hydraulic models were established to describe the flow patterns of the EMBR, and tracer experiments were executed to verify these models. Moreover, the detailed flow patterns of the EMBR were visualized by performing a two-dimensional (2-D) unsteady CFD model simulation. In addition, a mathematical model was developed to reveal the distribution of dissolved oxygen (DO) in the cathode of the EMBR. Afterward, the relationship between the reactor performance, which was assessed in terms of current generation, and the hydrodynamics of EMBR was further elucidated.

## Results and Discussion

### Hydraulic performance at various HRTs

**Figure 1:**
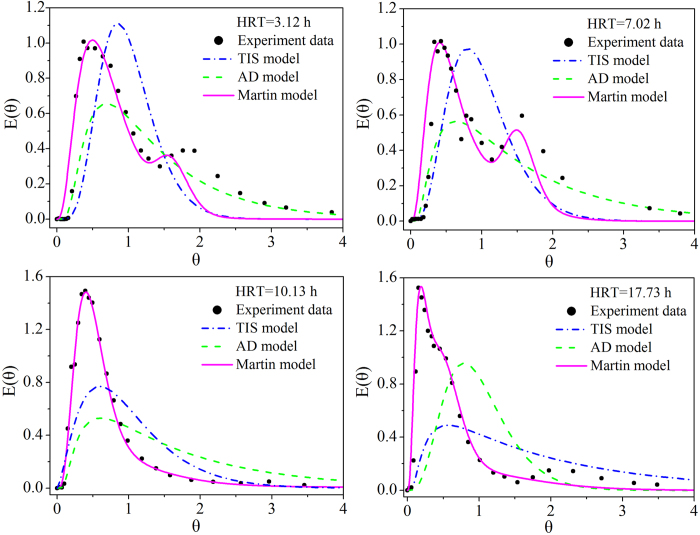
RTD curve and simulation results with the three models at various HRTs.

As shown in Fig. 1, two or three peaks are observed from each RTD curve at four different HRTs; therefore, a short circuiting stream or preferential flow existed in the EMBR because water leaked from different locations of graphite felt in the cathode and produced different channels of water from the inlet to the outlet in the EMBR^19^. Moreover, a long tail appeared in each RTD curve, especially at HRT values of 3.12 and 7.02 h, to imply that stagnant or dead zones were present in the EMBR and the release of the tracer Li^+^ was slow with flow stream in these regions^20^. In the EMBR, most of the anode chamber was taken up by the graphite felt, generating numerous micro zones with little mixing with main flow in the reactor, a portion of these spaces likely acted as transient storage or dead zones and therefore contributed to the tailing of the RTD curves.

### Comparison of the hydraulic models

Both TIS and AD models are widely used to simulate the hydrodynamics of the non-ideal flow reactors^21^, while Martin model could give better description on the preferential flow or short circuiting in a reactor^19^,^22^. Therefore, these three hydraulic models were selected to describe the hydrodynamic properties of the EMBR for comparison in this study.

As shown in Fig. 1, the Martin model generated better simulation results from the experimental data in each RTD curve compared to the others. In other words, neither the TIS nor AD models could characterize the hydrodynamics of the EMBR effectively. Moreover, the simulation reveals that three parallel sub-flows were involved in the Martin model for each HRT. Therefore, the water could flow primarily through the EMBR in three different channels, and each channel could be characterized by the TIS model. As shown in Table 1, the number (*N*) of CSTR in each strand of the Martin model was not below 3.4 at four HRTs. As discussed above, the degree of back-mixing in the reactor is insignificant if the value of *N* exceeds 3.01. Therefore, the flow pattern of each strand in the EMBR was similar to a plugged flow.

The dead volume of the EMBR at various HRTs estimated with both the Martin model and an RTD analysis are summarized in Table 1. The values of dead volume are almost identical at each HRT for the two methods, further implying that the Martin model could accurately simulate the hydrodynamic performance of the EMBR. In addition, Table 1 also shows that the dead zone became larger at higher or lower HRT in the EMBR. The liquid had a larger velocity at a shorter HRT; therefore, a vortex flow could form at the corner, possibly generated the higher values for the dead zones in the EMBR. However, a dead corner or stagnant zone would emerge in the reactor if the HRT was too long, possibly generating a larger dead zone in the EMBR.

The porous graphite felt was padded into the anode as the electrode material and dispersed unevenly inside, which resulted in a heterogeneous mixing in the EMBR. Although TIS and AD models are pervasive, however, both of them were developed based on the uniform dispersion degree in a reactor and thus wasn’t able to well simulate the heterogeneous mixing in the EMBR. Moreover, both TIS and AD models gave little information about dead volume and short circuiting of the EMBR. On the contrary, Martin model could describe the heterogeneous mixing in the reactors by adopting a number of separate strands, and therefore shown a better simulation performance on the hydrodynamics of the EMBR compared to TIS and AD ones.

### Flow pattern visualization through CFD simulation

In order to conduct CFD simulation, the permeability of both graphite felt and non-woven cloth in the EMBR was initially estimated through the porous media model. A porous media model does nothing but add a momentum sink to the governing momentum equations, generating pressure gradients in the porous media^23^. The momentum sink term in the model for simple homogeneous porous media can be described as:


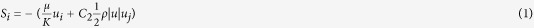


where *S*_*i*_ is the momentum source, and *K* and *C*_*2*_ are the permeability and inertial resistance, respectively. In our study, the inertial resistance of both the graphite felt and non-woven cloth could be treated as zero because water flowed slowly within the entire EMBR. In addition, all of the fibers in the graphite felt and non-woven cloth were treated as though they were randomly located in planes running parallel to gravity. Therefore, the permeability of the graphite felt and non-woven cloth could be determined using Eq. (2) according to a previous report:^24^


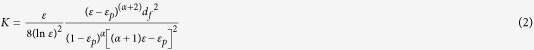


where *ε* is the porosity, *d*_*f*_ is the fiber diameter, and *ε*_*p*_ and *α* are constants that depend on the arrangement of the fibers. Values of 0.11 and 0.52 were suggested for *ε*_*p*_ and *α* for parallel permeability *K*_*P*_, while 0.11 and 0.785 were used for normal permeability *K*_*N*_^24^. Therefore, the estimated *K*_*P*_ and *K*_*N*_ values were 4.82 × 10^−10^ and 3.35 × 10^−12^ m^2^ for the graphite felt and 1.47 × 10^−14^ and 9.49 × 10–15 m^2^ for non-woven cloth, respectively.

**Figure 2:**
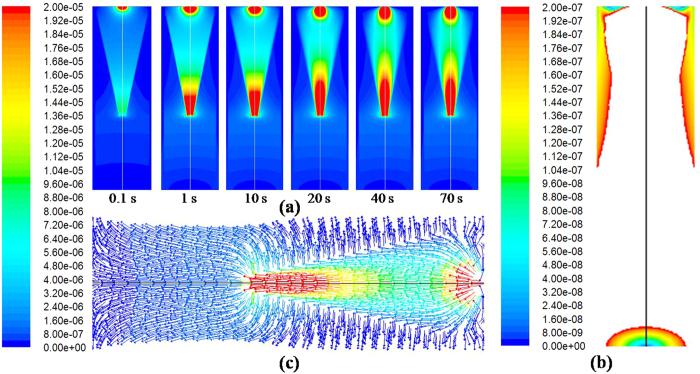
Transient model prediction for the EMBR at HRT = 3.12 h: (**a**) liquid velocity magnitude contour at 0.1, 1, 10, 20, 40 and 70 s; (**b**) liquid velocity vector field at 40 s; (**c**) approximate position of the dead zone in the EMBR (colorful zones).

Based on the above estimated values, a transient CFD simulation was performed over 70 s at each HRT, and the results show that the fraction of the liquid volume was always 100% in the entire EMBR. Specifically, no air bubbles could enter into the reactor during the simulation process, and therefore the velocity magnitude contours and vector fields of the mixture only the covered liquid phase in the EMBR region. When using an HRT of 3.12 h as an example, the variations in the liquid contour of velocity magnitude in the EMBR is shown in Fig 2a. The similar contours at 40 and 70 s imply that the fluid flow in the EMBR region had already reached a steady state by 40 s. Additionally, the flow velocity in the graphite felt zone was much lower than that in the non-graphite felt zones because the graphite felt imposed flow resistance on the water stream, causing a momentum loss in the water stream (Fig. 2a). Vesvikar and Al-Dahhan reported that the region where the flow velocity is less than five percent of the maximum among simulation zones could be considered the dead zone^25^. Consequently, the dead zones of the EMBR are located primarily at the bottom and upper outer regions of the reactor, as shown in Fig. 2b. Based on the velocity vector field at 40 s in Fig. 2c, the liquid flowed regularly with less back mixing or mixing with others in the anode without graphite felt, indicating that the flow pattern was closed to generate a plug flow in this area. However, the main flow of the stream would disperse to numerous branches after it entered the graphite felt zone of the anode and then discharge from different locations in the graphite felt in the cathode. This behavior would allow the generation of various channels of liquid from inlet to outlet in the EMBR, indicating the possibility of existing preferential or circuitous flow in the reactor.

### Relationship between current generation and hydrodynamics

The simulated DO distributions in the cathode of the EMBR at various HRTs are shown in Fig. S1. The DO concentration should have decreased when increasing the distance from the outside surface of the cathode at each HRT. However, a high flow velocity of liquid at a short HRT produced a low DO concentration in the cathode because the DO diffused in a direction opposing the flow of water in the EMBR.

**Figure 3:**
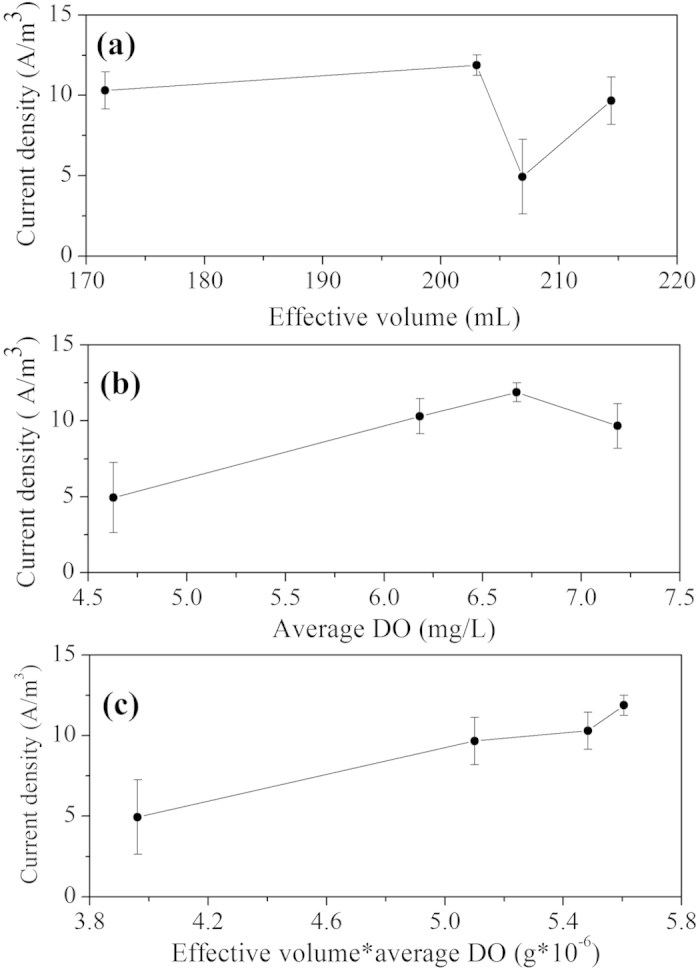
Variations in the current density relative to the (**a**) effective volume of the EMBR, (**b**) average DO concentration in the cathode, and (**c**) effective volume of the EMBR*average DO concentration in the cathode.

The existence of dead zones will decrease the effective volume in bioreactors, affecting the extent of the biochemical reactions. However, the oxygen transfer process is critical for air-cathode MFCs because it can determine the rate of the oxygen reduction reaction. Fig. 3 shows the variations in the current density in the EMBR with the effective volume of the reactor and average DO concentration in the cathode and multiplier. A positive relationship was found only between the current density and multiplier of two factors rather than a single one in the EMBR. Therefore, the power production in the EMBR was simultaneously determined through the effective volume of the reactor and the DO concentration in the cathode, which were both strongly affected by the hydrodynamics of the reactor, as discussed above. The DO had a lowest concentration because it had the highest flow velocity at HRT = 3.12 h (Fig. S1), therefore, the electricity generation in the EMBR was seriously limited, resulting in a minimum current density of 4.7 A/m^3^. Longer HRTs would produce higher DO concentrations in the cathode and may be beneficial for electricity production, but the dead volume of the reactor would increase and thus reduce the capability for electricity production in the EMBR (Table1). At HRT = 17.73 h, the ratio of the dead zones (29.3%) was significantly higher compared to the other HRTs, generating a lower current density of 9.2 A/m^3^ in the EMBR. These results demonstrate that the hydrodynamic characteristics of the reactor are critical for the performance of the EMBR. An optimized HRT could reduce the dead volume of the reactor while also providing an appropriate DO concentration in the cathode, thereby promoting electricity production in the EMBR.

In conclusion, the revealed hydrodynamic characteristics of the EMBR would help us know the degree of mixing as well as the value and location of dead zones, which will be conducive to improvement of the EMBR structure. Moreover, after an analysis on the relationship between the revealed hydrodynamic properties and the performance of the EMBR, it has been confirmed that HRT had a significant impact on the current generation, and thus it is imperative to choose an optimized HRT for the operation of the EMBR.

## Methods

### EMBR structure and operation

The schematic diagram of EMBR is shown in Fig. S2a. Non-woven cloth with 75% porosity and 50-mm pores supported by a perforated polyvinyl chloride (PVC) tube was placed between cathode and tubular anode chambers. The non-woven cloth could separate anodic and cathodic electrodes thus avoiding short circuit. Graphite felt with 93.5% porosity and 150-μm pores (Sanye Carbon Co., China) was served as the electrode both in anode and cathode. In the anode chamber (height 20 cm, diameter 4.5 cm), the graphite felt with a total volume area of 197.6 mL was rolled into a swirl so that the water from the top of the EMBR could flow more uniform, and the working volume of the anode chamber was 247.1 mL. The cathode of the EMBR was composed of a graphite felt with a projected surface area of 294 cm^2^ that wrapped around the non-woven cloth. Besides, electrodes were connected to the circuit through titanium wires across an external resistor (100 Ω), the reactor was operated in a continuous-flow mode at around 25oC^9^,^26^. The electrons and protons were generated when consuming substrate by the electrochemically active bacteria at the anode, while at the cathode electrons from the anode was reacted with oxygen in the air and protons diffused from anode.

The electrochemical reactor usually consists of anode and cathode as well as the separator membrane. In the EMBR, the membrane was replaced by the non-woven cloth, which could be used as not only a separator but also a filter for treated water from anode. Besides, the bacteria was inoculated both in the anode and cathode of the EMBR to consume organic and inorganic matters. Additionally, both anodic and cathodic pHs of the EMBR were kept at a natural value because of bacteria inside, and moreover the operational HRT would not be too short due to the biofilm formation in both compartments.

### Tracer test

Tracer test is often used to produce RTD curves in different systems. Lithium-ion has been widely used as the tracer not only due to its simple measurement procedure, low detection limit and low background, but also because it does not volatilize, precipitate or absorb in the reactor. Based on lithium-ion tracer experiments, Capela *et al.* obtained information about mixing degree and non-effective volume in a full-scale anaerobic contact reactor^27^, while Wang *et al.* found that dosing of ferrous salts at two different points would bring two distinct effects on the reactor in a pilot scale membrane reactor^28^. Besides, lithium tracer has also been applied to provide RTD curves in an electrochemical system^29^.

In this study, a 2 g/mL aqueous Li_2_SO_4_ solution was used as the tracer in the experiments, and the pulse input method was adopted through the use of deionized water as the carrying fluid. One milliliter of the Li_2_SO_4_ solution was injected into the flowing water of the EMBR over 0.5 s, producing an average Li^+^ concentration of 10 mg/L in the entire reactor. As summarized in Table S1, four different hydraulic retention times (HRTs) were investigated by controlling the flow rate of the EMBR with a peristaltic pump (Longer Co., China). The samples were collected from the EMBR effluent during injection before stopping after fourfold HRTs^30^. The Li^+^ concentration in the sample was measured through flame emission spectroscopy (Vario 6, Analytik Jena AG, Germany) above 670.8 nm according to the Standard Methods^31^.

### RTD analysis

The exit age-distribution function *E*(*t*) can be expressed as follows:


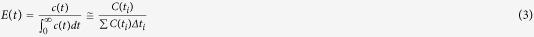


where c(*t*) is the tracer concentration at the outlet of the reactor^32^. The mean residence time *t*_*m*_ is calculated as follows:


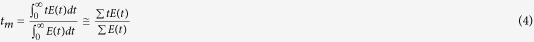


when comparing the mixing performance of the differently sized flow systems, all of the parameters should be normalized versus normalized time *θ*:


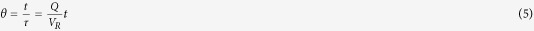


where *τ* is the hydraulic residence time, *V*_*R*_ is reactor volume, and *Q* is volume flow rate. Therefore, the normalized RTD function *E*(*θ*) can be presented as follows:


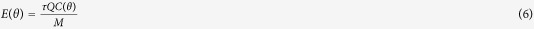


where *M* is the total amount of tracer. The dimensionless variance 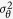, which characterizes the degree of back-mixing degree in the reactors, is defined as follows:


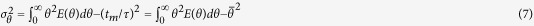


A 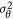 value of 0 indicates an ideal plug flow, while 1 indicates an ideal mixture; when the flow pattern is not ideal, the 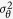 value is between 0 and 1.

A dead zone (*V*_*d*_) in an area where fluids move slowly or stagnate, and these areas often appear at the corners of a reactor. *V*_*d*_ can be calculated as follows:


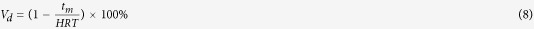


### Development of hydraulic models

#### Tank-in-series (TIS) model

**Figure 4:**
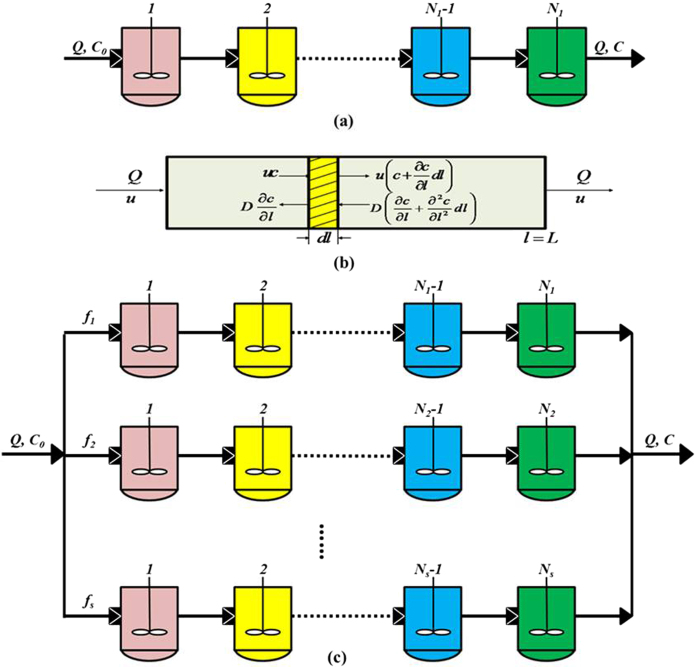
Block diagram of the (**a**) TIS, (**b**) AD and (**c**) Martin models: *Q* is the volume flow rate; for the Martin model, *f* represents volume flow rate fraction of each strand, and the dead volume (*V*_*d*_) = total volume (*V*_*t*_) - 1st strand region (*V*_*1*_) - 2nd strand region (*V*_*2*_) - … - *s*th strand region (*V*_*s*_).

As shown in Fig. 4a, this model consists of a series of equivalent, continuous stirred tank reactors (CSTRs), and the number (*N*) of tanks can be calculated as follows:


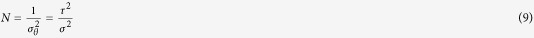


*N* is a criterion that predicts the flow pattern in a reactor^32^. The flow pattern of the reactor is closer to a complete mixing flow if *N* remains below 3.01; in all other cases, the pattern is more similar to a plug flow^21^. The exit age-distribution function *E*(*θ*) of the TIS model is given as follows:


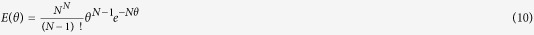


#### Axial dispersion (AD) model

A schematic diagram of axial dispersion model for the EMBR is shown in Fig. 4b, and its exit age-distribution function *E*(*θ*) can be described as


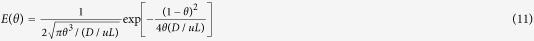


Where *D* is the axial dispersion coefficient, and *D/uL* is defined as the Peclet number (*Pe*), which represents the ratios of mass transport attributed to advection and dispersion, respectively^32^. The Peclet number is also a criterion that predicts the degree of back-mixing in a reactor, and the relationship between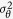 and *Pe* can be described as follows:


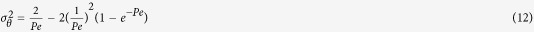


#### Martin model

This model can evaluate the short circuiting stream or preferential flow in reactors while determining the dead volume accurately^19^. A schematic diagram of this model for the EMBR is shown in Fig. 4c, consisting of several parallel strands; each strand is considered as the number of CSTRs in series. Therefore, the following equation was obtained based on the mass balance:


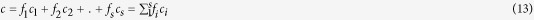


where *c*_*i*_ and *c* are the tracer concentration at each strand outlet and total outlet, respectively, and *c*_*i*_ can be calculated based on the TIS model:


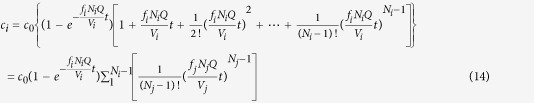


where *f*_*i*_*, N*_*i*_ and *V*_*i*_ represent the volume flow rate fraction, the number of tanks and the volume of the *i*th strand, respectively. Therefore, the Li^+^ concentration at the total outlet *c* and the RTD function *E*(*t*) can be expressed as follows:


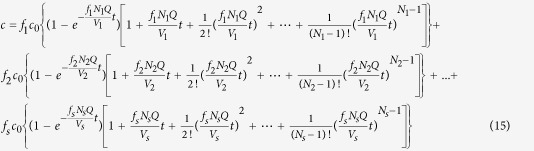



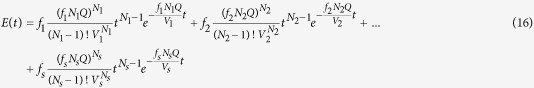


Here (*N*_*i*_*-1*)! is replaced by Gamma distribution Γ(*N*_*i*_) such that Eq. (16) would fit for all positive integer values^22^. Therefore, the dimensionless form of Eq. (16) could be given as follows:


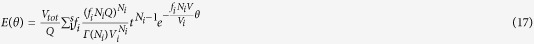


The value of *N*_*i*_ can be evaluated based on:


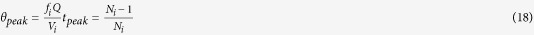


where *t*_*peak*_ and *θ*_*peak*_ are the time and dimensionless time of the maximal tracer concentration at each strand outlet, respectively.

In this study, the Microsoft EXCEL built-in solver^®^ was used to determine the values of various parameters in each model to reach the best correlation between the experimental data and the simulation results.

### CFD simulation

A 2-D axisymmetric unsteady CFD simulation was used to investigate the hydrodynamics of the EMBR through commercially available CFD software called FLUENT. The Gambit pretreatment software was chosen to divide grids in the simulated region, which was treated as a 2-D plane because an axisymmetric space was adopted for the simulation in this study, as shown in Fig. S2b. The detailed boundary conditions of this simulation are provided in the Supporting Information (SI). The Volume of Fluid (VOF) model, which is an Euler-Euler approach, was applied to address the multiple phases: gas and water^33^. In the VOF model, two or more phases share a single set of conservation equations, and the volume fraction of each phase is tracked in each computational cell throughout the domain. The conservation equations for mass and momentum in the VOF formulation are defined as follows, respectively:


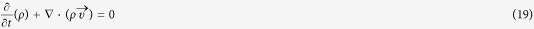



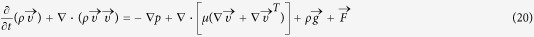


The density (*ρ*) and viscosity (*μ*) in a two-phase system can be estimated using Eq. (21) and Eq. (22):


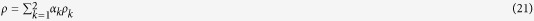



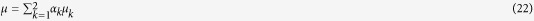


where *α*_*k*_ is the volume fraction of phase *k*. The sum of the volume fractions in all phases should equal one in each control volume. The interface between the two phases in the VOF model is tracked by solving the continuity equation in the volume fraction equation:


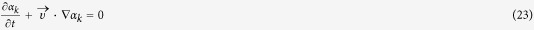


### Modeling the DO distribution in the cathode

A mathematical model was built to investigate the DO distribution in the cathode, and the schematic diagram of this model is shown in Fig. S3. The cube in Fig. S3 was considered part of the graphite felt, and the directions of the DO diffusion and water flow were horizontal but opposing. Moreover, the flow velocity of water was assumed equal along the entire piece graphite felt. Therefore, one-dimensional conservation laws were established for the DO distribution in the cathode:


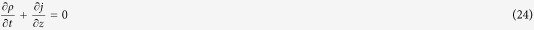



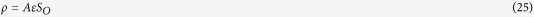



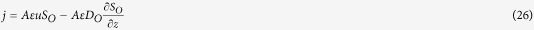


where 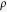 is the amount of conserved quantity per unit thickness of graphite felt, *j* is the amount of oxygen transported per unit time, *A* denotes the cross area of the graphite felt, *u* is the flow velocity in the graphite felt, and *D*_*o*_ is the oxygen diffusion coefficient in water (2.2 × 10^−9^ m^2^/s at 20 ^o^C). Substituting Eq. (25) and Eq. (26) into e Eq. (24) yields:


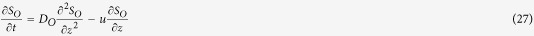


The boundary conditions are as follows:


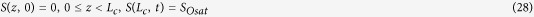


where *L*_*c*_ is the thickness of the graphite felt, and *S*_*Osa*t_ is the saturated solubility of oxygen in water (8 mg/L at 20 ^o^C). MATLAB program was executed to obtain the numerical solution of Eq. (27).

## Author Contributions

Y.Z.W. carried out the experiments, developed the models analyzed the data, and wrote the paper; Y.K.W. carried out the experiments and analyzed the data; C.S.H., H.Y.Y. and J.Y.S. analyzed the data; G.P.S. and H.Q.Y. designed the experiments and analyzed the data; Y.M. designed the experiments, analyzed the data, and wrote the paper.

## Additional Information

**How to cite this article**: Wang, Y.-Z. *et al.* Hydrodynamics of an Electrochemical Membrane Bioreactor. *Sci. Rep.* **5**, 10387; doi: 10.1038/srep10387 (2015).

## Supplementary Material

Supplementary Information

## Figures and Tables

**Table 1:** Estimated values of the different parameters for the three models at various HRTs.

**HRT**	**TIS model**	**AD model**	**Martin model**										***V***_***dead***_ **(%**,**1*****-t***_***m***_***/HRT***)
	***N***	***Pe***	***f***_***1***_	***V***_***1***_	***N***_***1***_	***f***_***2***_	***V***_***2***_	***N***_***2***_	***f***_***3***_	***V***_***3***_	***N***_***3***_	***V***_***dead***_**(%)**	
3.12	5.1	9.15	0.3	37.5	3.9	0.56	113.9	4.2	0.14	55.3	55	14.48	13.51
7.02	5	8.84	0.6	77.8	3.9	0.22	68.9	7.6	0.18	67.5	58	11.37	12.09
10.13	2.5	3.62	0.76	92.1	4.7	0.2	62.6	5	0.04	53.6	4.8	15.60	16.25
17.73	4.7	8.34	0.38	21.4	4	0.43	60.9	7.5	0.19	87.9	3.8	29.70	31.53
