# Supplementary material for: Hydrodynamics of an Electrochemical Membrane Bioreactor
Source: Sci Rep. 2015 May 22;5:10387. doi: 10.1038/srep10387 (PMC4441134; doi:10.1038/srep10387)
Supplement: Supplementary Information [file srep10387-s1.doc]

**Supplementary Information for**

**Hydrodynamics of an Electrochemical Membrane Bioreactor**

Ya-Zhou Wang^a^, Yun-Kun Wang^a^, Chuan-Shu He^a^, Hou-Yun Yang^a^, Guo-Ping Sheng^a^, Jin-You Shen^b^, Yang Mu*^a,b^, Han-Qing Yu^a^

^a^CAS Key Laboratory of Urban Pollutant Conversion, Department of Chemistry, University of Science & Technology of China, Hefei, China

^b^Jiangsu Key Laboratory of Chemical Pollution Control and Resources Reuse, Nanjing University of Science and Technology

***Corresponding author:**

Dr. Yang Mu, Fax: +86 551 63607907; Email: [yangmu@ustc.edu.cn](mailto:yangmu@ustc.edu.cn)

**Boundary conditions for CFD simulation**

In this study, the convergence and the floating point error would occur for 3-D and 2-D simulations respectively when the edge of graphite felt at the cathode was considered as the outlet in the CFD simulation. Therefore, an alternative outlet was chosen in the CFD model and moreover this outlet was far from the EMBR in the simulation region in order to avoid the appearance of reversed flow, as shown in Figure S1(b). The simulation zone was patch on air at *t*=0 s excluding EMBR region. The falling of liquid which oozed from graphite felt at the cathode could be simulated by using the VOF model. In addition, both non-woven cloth and graphite felt were simulated by the porous media model because of the anisotropy of these two materials in this study.

| **Table S1. Operating conditions for tracer test** | | |
| --- | --- | --- |
| HRT(h) | Flow rate (mL/h) | Velocity of inlet (×10^-4^ m/s) |
| 3.12 | 77.50 | 10.96 |
| 7.02 | 34.44 | 4.86 |
| 10.13 | 23.85 | 3.37 |
| 17.73 | 13.63 | 1.92 |

**Figure S1**. Simulated DO concentration at different locations of graphite felt in the cathode (*d*: The distance from outside surface of graphite carbon at the cathode).


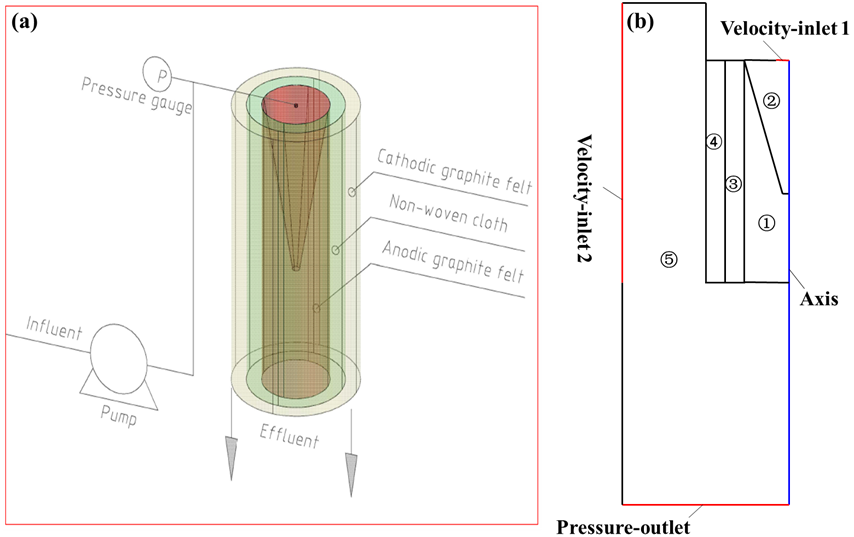


**Figure S2.** (a) Schematic diagram of the EMBR system; (b) overview of the simulation region for EMBR: (1) anodic graphite felt zone, (2) anodic zone without graphite felt, (3) cathodic non-woven cloth zone, (4) cathodic graphite felt zone, (5) air areas around EMBR; water into the anode from velocity-inlet 1, while air into the cathode from velocity-inlet 2.


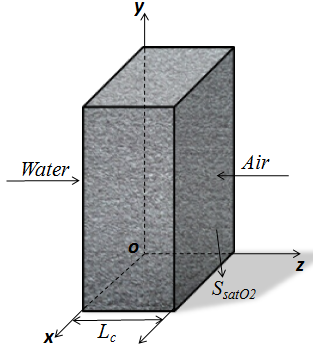


**Figure S3**. Schematic diagram for modeling DO distribution in the cathode of the EMBR.
